# Supplementary material for: Chromothripsis during telomere crisis is independent of NHEJ, and consistent with a replicative origin
Source: Genome Res. 2019 May;29(5):737–49. doi: 10.1101/gr.240705.118 (PMC6499312; doi:10.1101/gr.240705.118)
Supplement: Supplemental Material [file supp_gr.240705.118_Supplemental_file_1.zip › contigs/annotated_contigs/DB76/contig.2.DB76_length_660_mean_cov_9.18787878788.docx]

**DB76_length_660_mean_cov_9.18787878788**

```
CATGTAACTAGAATGCACCTGTACATGAATTTTAAAATGTCATACTGTGCTATTTTTCTTAATTCTGTAAGGGTAACAAGTAACTCAGT
 >chr10:74890858-74891182 - E=3e-183
AATGACTTAAAACTTGCTATATATTTTCAATACTAAGATAAAAGTTTTAACCAAACTTACAACAGGCACATTTTGGCAGTTAGTGTAAA

CACAACATTAACAAGCCATATGCCAGAAGATGTTTGCTTCCAAGGCCTTAGAGAAAATGACTGAACAAGGGCCTACATCCAAACTAATG

CTTCCCCCTTCCCCCTGATTTTTGACAGTTGACAGCTCATCAGTTTTCTTCTCAC|AC|CGGTGCTGGTCTGTGGCCTGTTAGGAACTG
                                                        >chr10:74935820-74936135 - E=9e
GGCTGCAGAGCAGGAGGTAAGTGGCAGGTGAGTGCATGAAGCTTCATCTGTATTTACAGCAGCTCCCCATGGCTCGCATTACCACCTGA
-178
GCTCTGCCTTCTGTCAGATCAGTGGTGGCATCAGATTCTCACAGGAGCACAAACTGCGCATGCAAGGGATCTAGGTTGTGCATTCCTTA

TGGGAATCTAATGCCTGATCATCTGTCACTGTCTCCCATCATCCCCGGATGGAACCATCTAGTTGCAGGAAAACAAGCTCAAGGCTCCC

ACTGATTCTACATTAT|AGATCGGAAGAGCACACGTCTGA
```
